# Circulating long non‐coding RNAs *NRON* and *MHRT* as novel predictive biomarkers of heart failure

**DOI:** 10.1111/jcmm.13101

**Published:** 2017-03-14

**Authors:** Lina Xuan, Lihua Sun, Ying Zhang, Yuechao Huang, Yan Hou, Qingqi Li, Ying Guo, Bingbing Feng, Lina Cui, Xiaoxue Wang, Zhiguo Wang, Ye Tian, Bo Yu, Shu Wang, Chaoqian Xu, Mingyu Zhang, Zhimin Du, Yanjie Lu, Bao Feng Yang

**Affiliations:** ^1^ Department of Pharmacology (the State‐Province Key Laboratories of Biomedicine‐Pharmaceutics of China, Key Laboratory of Cardiovascular Research, Ministry of Education) College of Pharmacy Harbin Medical University Harbin Heilongjiang China; ^2^ Department of Epidemiology and Biostatistics Public Health School Harbin Medical University Harbin Heilongjiang China; ^3^ Department of Cardiology the First Affiliated Hospital Harbin Medical University Harbin Heilongjiang China; ^4^ Division of Pathophysiology (the State‐Province Key Laboratories of Biomedicine‐Pharmaceutics of China and the Key Laboratory of Cardiovascular Research, Ministry of Education) Harbin Medical University Harbin Heilongjiang China; ^5^ Department of Cardiology the Second Affiliated Hospital Harbin Medical University Harbin Heilongjiang China; ^6^ Institute of Clinical Pharmacology the Second Affiliated Hospital Harbin Medical University Harbin Heilongjiang China; ^7^ Department of Pharmacology and Therapeutics Melbourne School of Biomedical Sciences Faculty of Medicine Dentistry and Health Sciences University of Melbourne Melbourne Australia

**Keywords:** heart failure, LncRNA, *NRON*, *MHRT*, plasma

## Abstract

This study sought to evaluate the potential of circulating long non‐coding RNAs (lncRNAs) as biomarkers for heart failure (HF). We measured the circulating levels of 13 individual lncRNAs which are known to be relevant to cardiovascular disease in the plasma samples from 72 HF patients and 60 non‐HF control participants using real‐time reverse transcription‐polymerase chain reaction (real‐time RT‐PCR) methods. We found that out of the 13 lncRNAs tested, non‐coding repressor of NFAT (*NRON*) and myosin heavy‐chain‐associated RNA transcripts (*MHRT*) had significantly higher plasma levels in HF than in non‐HF subjects: 3.17 ± 0.30 *versus* 1.0 ± 0.07 for *NRON* (*P *<* *0.0001) and 1.66 ± 0.14 *versus* 1.0 ± 0.12 for *MHRT* (*P *<* *0.0001). The area under the ROC curve was 0.865 for *NRON* and 0.702 for *MHRT*. Univariate and multivariate analyses identified *NRON* and *MHRT* as independent predictors for HF. Spearman's rank correlation analysis showed that *NRON* was negatively correlated with HDL and positively correlated with LDH, whereas *MHRT* was positively correlated with AST and LDH. Hence, elevation of circulating *NRON* and *MHRT* predicts HF and may be considered as novel biomarkers of HF.

## Introduction

HF is a major public health problem afflicting a large population (>25 million patients) in the world 1 and an intricate pathophysiological syndrome consequent to feeble cardiac contraction and inadequate blood ejection 2. The clinical manifestations of HF mainly arise from myocardial infarction (MI), hypertension, myocarditis and inherited cardiomyopathy 3, 4. Without successful intervention within a certain timeframe, HF can cause sudden cardiac death or severe disability, being the most devastating cardiovascular disease in terms of mortality, morbidity and the quality of life. One of the difficulties for timely treatment of HF is our current dearth of sensitive and specific biomarkers for early diagnosis of the malady. A number of clinically validated biomarkers such as cardiac troponin, natriuretic peptide, B‐type natriuretic peptide (BNP) and N‐terminal proBNP (NT‐proBNP) have been used in the diagnosis of HF 5, 6, 7, 8, 9, 10. Nonetheless, these traditional biomarkers have some limitations in defining the aetiology or prognosis of HF 5, 6, 7. For example, none of these markers are specific to HF, but their serum/plasma levels can rise in a number of other diseases such as cardiopulmonary disease, kidney failure and hepatic cirrhosis. Quest for more reliable biomarkers is therefore highly desirable. It is known that aberrant changes in the expression of multiple genes in myocardium are a major cause, as well as useful predictors of the pathologic remodelling in failing heart. Identification of such genes, particularly those that are highly sensitive and specific to HF, may be the key step towards reliable early prediction of HF.

Non‐coding RNAs (ncRNAs), including microRNAs (miRNAs) and lncRNAs, have recently been found to play important regulatory roles in the development and progression of cardiovascular diseases 11, 12, 13, 14. These RNAs have also been implicated in the diagnosis of cardiovascular diseases owing to the characteristic alterations of their circulating levels with different categories and grades of pathological processes. LncRNAs belong to a newly discovered class of functional mRNA‐like transcripts that lack significant open reading frames or protein‐coding capacity 14 and have emerged as an important player in cardiovascular diseases, including a number of cardiac‐specific or cardiac‐related lncRNAs such as *SRA*,* DIO3OS*,* SAF*,* NESPAS*,* MIAT*,* NRON*,* CARL*,* HCG22*,* FENDRR*,* MHRT*,* aHIF*,* ZFAS1* and *CDR1AS* 15, 16, 17, 18, 19, 20, 21, 22, 23, 24, 25, 26 (http://cmbi.bjmu.edu.cn/lncrnadisease). Recent research data have also suggested the roles of lncRNAs in HF 15, 16, 17, 18. Most prominently, circulating lncRNAs are exceptionally stable in the bloodstream and readily detectable in human subjects, such as in patients with cancers or acute kidney injury, implying that circulating lncRNAs might be a non‐invasive and rapid diagnostic tool for disease diagnosis and prognosis 15. However, studies on circulating lncRNAs for the prediction of HF have been sparse. A comprehensive study using microarray analysis compared expression alterations of lncRNAs in the heart, whole blood and plasma in a mouse model of acute HF 17. Their results revealed 32 differentially expressed lncRNAs with changes greater than twofold. Another study conducted with serum samples from HF patients suggested the potential of *LIPCAR* (the mitochondrial lncNA uc022bqs.1) to predict survival in patients with HF. Yet, none of these deregulated lncRNAs belong to the cardiac‐specific or cardiac‐related ones mentioned above.

This study was therefore designed to explore the possibility of the known cardiac‐specific and cardiac‐related lncRNAs in plasma samples from patients with HF as circulating biomarkers for HF. Quantitative RT‐PCR was employed to determine the plasma levels of the test lncRNAs. Our results identified *NRON* and *MHRT* as possible novel biomarkers for predicting HF.

## Materials and Methods

### Participants

Between February 2014 and January 2015, 104 HF patients and 109 non‐HF control participants presented to the First Affiliated Hospital, the Second Affiliated Hospital, the Third Affiliated Hospital and the Fourth Affiliated Hospital of the Harbin Medical University (Harbin, China). Diagnosis of HF and the criteria for inclusion of patients were as previously described in detail 27, 28 (see Supplementary Methods). The clinical characteristics of the study population are summarized in Table 1 and Tables S1 and S2.

**Table 1:** The demographic characteristics and HF‐relevant indicators in HF patients and non‐HF control participants

Characteristics	Non‐HF	HF	*P* value
Age			
*N* (missing)	60 (0)	72 (0)	0.8710
Mean (Std)	60.08 (11.97)	59.31 (11.19)	
Min, max	36, 88	28, 83	
Median	58	60.50	
Range	52~67.50	51~67	
Gender			
Male	37	47	0.6676
Female	23	25	
Total (missing)	60 (0)	72 (0)	
Hypertension			
Yes	17	39	0.2858
No	19	28	
Total (missing)	36 (24)	67 (5)	
Diabetes			
Yes	7	17	0.4480
No	29	48	
Total (missing)	36 (24)	65 (7)	
CHOL			
*N* (missing)	57 (3)	63 (9)	0.0344
Mean (Std)	4.72 (0.70)	4.40 (1.11)	
Min, max	3.24, 6.33	1.98, 8.84	
Median	4.69	4.31	
Range	4.33~5.21	3.71~5.03	
TG			
*N* (missing)	57 (3)	63 (9)	0.3930
Mean (Std)	1.32 (0.52)	1.49 (0.74)	
Min, max	0.66, 2.43	0.49, 3.65	
Median	1.16	1.26	
Range	0.91, 1.51	0.92~1.89	
HDL			
*N* (missing)	57 (3)	63 (9)	0.00739
Mean (std)	1.20 (0.23)	1.12 (0.30)	
Min, max	0.79, 1.88	0.79, 2.85	
Median	1.20	1.07	
Range	1.06~1.31	0.95~1.21	
LDL			
*N* (missing)	57 (3)	63 (9)	0.6399
Mean (Std)	2.84 (0.56)	2.95 (0.92)	
Min, max	1.70, 4.31	0.93, 6.36	
Median	2.86	2.75	
Range	2.46~3.23	2.28~3.44	
Glycemia			
*N* (missing)	57 (3)	67 (5)	0.13911
Mean (Std)	5.99 (1.90)	6.93 (4.25)	
Min, max	3.98, 14.20	2.89, 36.6	
Median	5.46	5.86	
Range	5.11~6.04	5.03~7.34	
ALT			
*N* (missing)	26 (34)	65 (7)	0.0737
Mean (Std)	23.27 (12.23)	30.49 (20.31)	
Min, max	11.00, 59.00	0.26, 108.00	
Median	21.50	25	
Range	15.00~27.00	17.00~35.00	
AST			
*N* (missing)	26 (34)	65 (7)	0.02176
Mean (Std)	20.73 (3.81)	28.14 (15.27)	
Min, max	14.00, 28.00	7.00, 92.00	
Median	20.50	24.00	
Range	18.00~23.00	19.00~32.00	
AST/ALT			
*N* (missing)	23 (37)	65 (7)	0.2975
Mean (Std)	1.06 (0.36)	1.39 (3.02)	
Min, max	0.50, 1.80	0.40, 25.00	
Median	1.00	0.85	
Range	0.80~1.30	0.70~1.20	
BUN			
*N* (missing)	55 (5)	67 (5)	<0.0001
Mean (Std)	5.76 (1.46)	7.36 (2.65)	
Min, max	3.30, 10.34	3.07, 18.04	
Median	5.50	6.94	
Range	4.53~6.85	5.64~8.77	
Cr			
*N* (missing)	57 (3)	68 (4)	0.00215
Mean (Std)	72.96 (16.64)	90.11 (34.60)	
Min, max	8.20, 103.60	6.31, 239.30	
Median	75.00	81.55	
Range	62.20~85.00	67.70~100.80	
Bun/Cr			
*N* (missing)	23 (37)	68 (4)	<0.0001
Mean (Std)	84.25 (24.31)	48.90 (40.44)	
Min, max	45.92, 159.08	0.05, 124.58	
Median	83.69	58.73	
Range	69.00~93.00	0.12~78.58	
UA			
*N* (missing)	56 (4)	68 (4)	<0.0001
Mean (Std)	316.21 (79.21)	422.88 (144.25)	
Min, max	156.40, 516.00	88.75, 799.70	
Median	304.30	387.15	
Range	257.10~374.30	320.45~532.95	
Co2CP			
*N* (missing)	52 (8)	48 (24)	0.4233
Mean (Std)	36.48 (47.05)	37.44 (67.67)	
Min, max	23.20, 281.00	10.00, 495.70	
Median	27.35	27.75	
Range	26.10~28.50	26.00~30.00	
NT‐proBNP			
*N* (missing)	0 (60)	65 (7)	NA
Mean (Std)		3786.62 (6091.64)	
Min, max		104.00, 35,000.00	
Median		2144.00	
Range		635.00, 3788.00	

CHOL, total cholesterol; TG, triglyceride; HDL, high‐density cholesterol; LDL, low‐density cholesterol; ALT, alanine aminotransferase; AST, aspartate aminotransferase; BUN, blood urea nitrogen; Cr, creatinine; UA, uric acid; Co2CP, carbon dioxide combining power; NT‐proBNP, amino‐terminal pro‐brain natriuretic peptide.

### Ethical approval of studies and informed consent

The study protocols and the procedures for handling human samples were approved by the Institutional Research Board of the Harbin Medical University (No.HMUIRB‐20140027). The written informed consents were obtained from all subjects recruited to our study.

### Collection and handling of human blood samples

Whole blood samples (1 ml per patient) were drawn from the study subjects *via* a direct venous puncture into the tubes containing sodium citrate. The human whole blood samples in sodium citrate vacuum tubes were kept at 4°C and then centrifuged at 2000 × g/min. at 4°C for 10 min. to obtain plasma samples.

### RNA extraction and quantitative real‐time reverse transcription (RT)‐polymerase chain reaction (qPCR)

Total RNA was extracted from the prepared plasma samples using Trizol LS reagent (Invitrogen, Carlsbad, California, USA) according to the manufacturer's instructions. In brief, each plasma sample (0.25 ml) was mixed well with 1 ml Trizol reagents in a tube. Chloroform (0.2 ml) was added into the sample and shaken vigorously by hand for 15 sec. The sample was incubated at room temperature for 5 min. and then centrifuged at 12000 × g/min. at 4°C for 15 min. The supernatant was transferred to a new tube, and an equal volume of isopropanol was added to the aqueous phase. After mixing and incubation at room temperature for 10 min., the sample was again centrifuged at 12000 × g/min. at 4°C for 10 min. After removal of the supernatant, the pellet was washed with 1 ml of 75% ethanol for the initial homogenization. Then, the sample was centrifuged at 10,600 r.p.m./min. at 4°C for 5 min. The RNA pellet was dissolved in DEPC water. The quality of our RNA samples was first measured by NanoDrop ND‐8000 (Thermo Fisher Scientific, Waltham, MA, USA). To ensure the RNA/DNA ratio 1.8–2.0. Then, the integrity of the RNA samples was assessed by standard denaturing agarose gel electrophoresis and confirmed by discrete 28 s and 5 s bands without smear.

The SYBR Green PCR Master Mix Kit (cat#: 4367659, Life technology, USA) was used for qPCR for relative quantification of lncRNAs (see Supplementary Materials online for detail). The qPCR primer pairs used in our study are listed in Table S4 online.

### Statistical analysis

Categorical data are presented as count and percentile. Continuous variables are described as mean ± S.E.M. (standard error of measurement), min, max, median or interquartile range, as specified in the data descriptions. The statistical analyses are described in detail in supplementary methods. All analyses were carried out with SAS 9.1 (Serial No. 989155) except that ROC was carried out with SPSS v17.0 software. The significant level was set at 0.05, and two‐tailed *P* values <0.05 were considered statistically significant.

## Results

### Clinical characteristics of the study population

Plasma samples were collected from a total of 104 HF patients and 109 control participants for measuring lncRNAs. To have more rational comparisons between HF and control participants, we filtered the plasma samples based upon the clinical or demographic characteristics of the patients recruited. We identified 32 HF patients and 49 control participants who did not have matched clinical or demographic characteristics between the two groups, and we therefore discarded these samples leaving 72 HF patients and 60 control participants for detailed statistical analysis. Of the 72 HF patients, 65 had an elevated NT‐proBNP at enrolment during the study period. Table 1 shows the clinical and demographic characteristics of the patients enrolled in this study (also see Tables S1 and S2 online for the complete data sets of all 104 HF patients and 109 control participants). There were no age and gender differences between the test patients and control participants, nor was any difference in blood pressure.

### Reciprocal changes in *NRON* and *MHRT* blood levels in AMI patients

**Figure 1:**
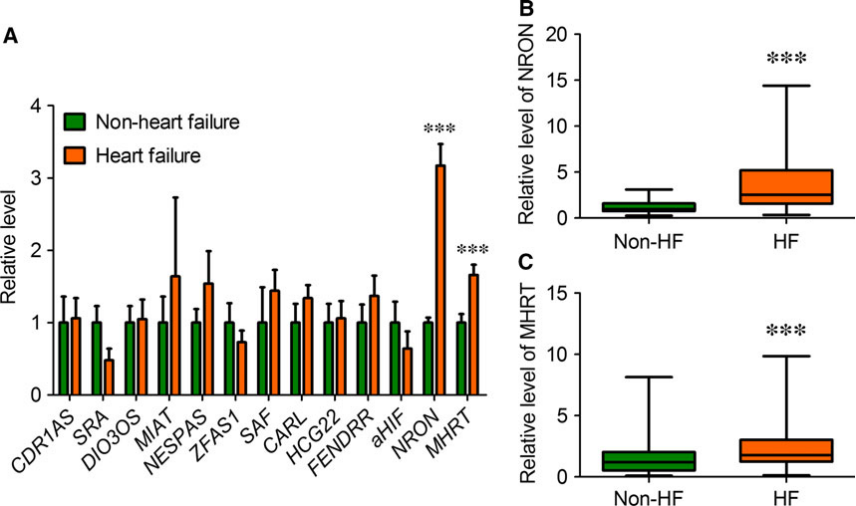
Changes in circulating lncRNA levels in patients with HF relative to non‐HF control participants. (**A**) Circulating levels of lncRNAs were determined by quantitative real‐time RT‐PCR (qPCR) with the plasma samples prepared from HF patients and non‐HF control participants. Note that only *NRON* and *MHRT* demonstrated significant differences between patients HF and non‐HF control participants. Data are presented as mean ± S.E.M. ***P <* *0.0001, *n* = 72 for HF and *n* = 60 for non‐HF control participants. (**B** & **C**) Box plot of plasma *NRON* and *MHRT* levels, respectively, providing a non‐parametric illustration of numerical data displaying the degree of dispersion (spread), skewness in the data (asymmetry of distribution) and outliers, without making any assumptions of the underlying statistical distribution. ***P <* *0.0001, *n* = 72 for HF and *n* = 60 for non‐HF control participants.

Our initial quantitative real‐time RT‐PCR (qPCR) analysis included 13 known cardiac‐specific or cardiac‐related lncRNAs: *SRA*,* DIO3OS*,* SAF*,* NESPAS*,* MIAT*,* NRON*,* CARL*,* HCG22*,* FENDRR*,* MHRT*,* aHIF*,* ZFAS1* and *CDR1AS*. As illustrated in Figure 1A, of 13 lncRNAs tested, only two, *NRON* and myosin heavy–chain‐associated RNA transcripts (*MHRT*), demonstrated significant differences in plasma samples between HF and non‐HF. Specifically, the circulating level of *NRON* was significantly higher in HF than in non‐HF subjects (3.17 ± 0.30 *versus* 1.0 ± 0.07; *P *<* *0.0001) (Fig. 1B and C; Table 2). Similarly, the plasma level of *MHRT* was also markedly elevated in HF (1.66 ± 0.14) relative to that in non‐HF subjects (1.00 ± 0.12; *P *<* *0.0001). The median Ct value for *NRON* was 26.3 by 40 cycles of qPCR with standard deviation of 2.2, and the median Ct value for *MHRT* was 27.0 with standard deviation of 1.6, indicating that these two lncRNAs are fairly abundant in plasma.

**Table 2:** Statistical analysis of the circulating *NRON* and *MHRT*

LncRNA	Non‐HF	HF	*P* value
***MHRT***			
*N* (missing)	60 (0)	72 (0)	<0.0001*
Mean (Std)	1.0 (0.89)	1.66 (1.2)	
Min, max	0.06, 5.85	0.10, 7.08	
Median	0.86	1.27	
Range (Q1, Q3)	0.38~1.43	0.90~2.15	
***NRON***			
*N* (missing)	60 (0)	72 (0)	<0.0001*
Mean (Std)	1.0 (0.54)	3.17 (2.58)	
Min, max	0.09, 8.14	0.13, 9.86	
Median	1.20	1.77	
Range (Q1, Q3)	0.54~1.26	1.26~2.99	

*P* values are for comparisons between HF patients *versus* non‐HF control participants. * *P* < 0.001 vs. Non‐HF.

Similar elevations of the circulating levels of *NRON* and *MHRT* were consistently observed when all plasma samples (104 HF patients and 109 control participants) were included in our analysis (Table S3 online).

### Evaluation of circulating *NRON* and *MHRT* as new biomarkers for HF

**Figure 2:**
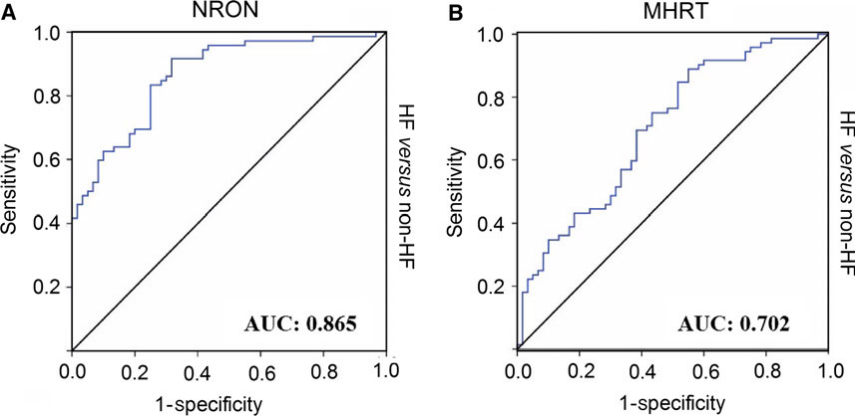
Receiver operator characteristic analysis of circulating *NRON* and *MHRT* for predicting HF. The area under ROC curve was determined to evaluate the predictive power of circulating *NRON* (**A**) and *MHRT* (**B**) levels for HF using non‐HF participants as control.

Having established that *NRON and MHRT* are present in the peripheral circulation and their plasma levels are anomaly altered in HF patients, we sought to determine the potential utility of circulating *NRON and MHRT* as diagnostic biomarkers of HF. To this end, ROC analysis was performed to evaluate the predictive power of circulating *NRON* and *MHRT* alone for HF. Our results showed that the area under ROC curve was 0.865 (95% CI = 0.805~0.926) for *NRON* alone (Fig. 2A), 0.702 (95% CI = 0.612~0.791) for *MHRT* alone (Fig. 2B).

The univariate analysis with logistic regression showed that the odds ratios (OR) were 4.505 (95% CI: 2.393~8.478) for *NRON* (*P *<* *0.0001), and 1.701 (95% CI: 1.225~2.363) for *MHRT* (*P *=* *0.0015) between HF and non‐HF (Table 3).

**Table 3:** Univariate regression analysis for the association of *NRON* and *MHRT* with demographic characteristics between HF patients and non‐HF control participants

Variable	B	S.E.	λ^2^	*P*	OR	95% CI	
						Low	High
*NRON*	1.5051	0.3226	21.7608	<0.0001	4.505	2.393	8.478
*MHRT*	0.5313	0.1676	10.0523	0.0015	1.701	1.225	2.363
Age	−0.00592	0.0153	0.1504	0.6982	0.994	0.965	1.024
Gender	0.1558	0.3630	0.1843	0.6677	1.169	0.574	2.381
HDL	−1.1380	0.7586	2.2509	0.1335	0.320	0.072	1.417
LDL	0.1928	0.2424	0.6326	0.4264	1.213	0.754	1.950
TG	0.4278	0.2985	2.0546	0.1518	1.534	0.855	2.754
CHOL	−0.3756	0.2072	3.2842	0.0699	0.687	0.458	1.031

The multivariate logistic regression analysis further verified *NRON* and *MHRT* as independent predictors for HF (Table 4): The OR values were 3.377 (95% CI: 1.441~7.915) for *NRON* (*P *=* *0.0051) and 1.679 (95% CI: 1.068~2.639 for *MHRT* (*P *=* *0.0248) between HF and non‐HF (Table 4).

**Table 4:** Multivariate regression analysis for the association of *NRON* and *MHRT* with demographic characteristics between HF patients and non‐HF control participants

Variable	B	S.E.	λ^2^	*P*	OR	95% CI	
						Low	High
*NRON*	1.2170	0.4346	7.8412	0.0051	3.377	1.441	7.915
*MHRT*	0.5182	0.2308	5.0395	0.0248	1.679	1.068	2.639
Age	0.00199	0.0315	0.0040	0.9496	1.002	0.942	1.066
Gender	−0.6181	0.7135	0.7505	0.3863	0.539	0.133	2.182
HDL	7.6350	2.0206	14.2775	0.0002	>999.999	39.437	>999.999
LDL	6.1932	1.4468	18.3247	<0.0001	489.417	28.720	>999.999
TG	−6.3369	1.4537	19.0036	<0.0001	0.002	<0.001	0.031
CHOL	2.6408	0.7249	13.2708	0.0003	14.024	3.387	58.066

### Relation of *NRON* and *MHRT* to conventional prognostic markers

To further evaluate the usefulness of circulating *NRON* and *MHRT* as HF biomarkers, we tested whether their levels were correlated with cardiac risk factors, conventional HF markers and cardiac function parameters. The data summarized in Table 5 show that *NRON* was negatively correlated with HDL and positively correlated with LDL, whereas *MHRT* was positively correlated with AST and LDH (Table 6). Neither *NRON* nor *MHRT* was correlated with age, gender, diabetes mellitus, hypertension, smoking history, total cholesterol, triglyceride (TG), cardiac troponin I (cTnI), aspartate aminotransferase 29, creatine kinase (CK), creatine kinase‐myocardial band (CKMB), NT‐proNBP or cardiac function parameters.

**Table 5:** Spearman's rank correlation analysis for the association of *NRON* with cardiac risk factors, cardiac biomarkers and cardiac function parameters in HF patients

	*NRON*	
	Coefficient	*P*
Cardiovascular risk factors		
Age	0.02786	0.7512
Gender	0.13804	0.1145
Diabetes	0.09415	0.3490
Hypertension	−0.03081	0.7573
Smoking	0.15804	0.2319
HDL	−0.22658	0.0128
LDL	0.11300	0.2191
CHOL	−0.12143	0.1864
TG	0.04193	0.6493
Cardiac biomarkers		
cTnI	0.06742	0.6088
AST	0.09553	0.4085
LDH	0.53876	<0.0001
CK	0.14788	0.1905
CKMB	0.05194	0.7814
NT‐proBNP	0.10304	0.4141
Cardiac function		
E/A	−0.36332	0.1263
EF	−0.22937	0.0952
FS	0.02309	0.9071

cTnI, cardiac troponin I; CK, creatine kinase; CKMB, creatine kinase‐myocardial band; E/A, E, peak velocity of the early diastolic filling wave, A, peak velocity of the late diastolic filling wave; EF, ejection fraction; FS, fractional shortening.

**Table 6:** Spearman's rank correlation analysis for the association of *MHRT* with cardiac risk factors, cardiac biomarkers and cardiac function parameters in HF patients

	*MHRT*	
	Coefficient	*P*
Cardiovascular risk factors		
Age	0.03310	0.7063
Gender	0.16987	0.0515
Diabetes	0.11410	0.2559
Hypertension	−0.00328	0.9738
Smoking	−0.00405	0.9757
HDL	−0.14948	0.1032
LDL	−0.07218	0.4334
CHOL	−0.10753	0.2424
TG	−0.01565	0.8653
Cardiac biomarkers		
cTnI	0.08386	0.5241
AST	0.35285	0.0016
LDH	0.43344	<0.0001
CK	0.02483	0.8270
CKMB	0.23464	0.2039
NT‐proBNP	0.07810	0.5363
Cardiac function		
E/A	0.15182	0.5350
EF	−0.14227	0.3048
FS	0.11656	0.5547

## Discussion

In the present study, we analysed the levels of a selected set of lncRNAs in the plasma samples of HF patients for their potential as biomarkers for the diagnosis of HF. These lncRNAs were selected for our study because they have been documented to play important roles in shaping developmental process of the heart and in the pathogenesis and progression of cardiac diseases 15, 16, 17, 18, 19, 20, 21, 22, 23, 24, 25, 26. Our results identified two lncRNAs, *NRON* and *MHRT* out of 13 known cardiac‐relevant lncRNAs examined, as promising candidate biomarkers for HF in the light of the significant elevations of their circulating levels in HF patients relative to non‐HF control participants and the close correlation between the circulating levels of *NRON* and *MHRT*.

### Published studies on circulating LncRNAs as HF biomarkers

*NRON* (Non‐coding RNA repressor of NFAT) is enriched in muscles (including cardiac muscle), placenta, spleen, thymus and lymph nodes and has been denoted as a repressor of the nuclear factor of activated T cells (NFAT) by influencing its nuclear trafficking 30, 31. NFAT is known to be a critical protein in the regulation of intracellular Ca^2+^ homoeostasis and of gene expression as a transcription factor in the heart, and its expression and activity are tremendously increased in HF 32, 33. It is conceivable that by regulating NFAT, *NRON* can participate in the genesis and development of HF. Yet, such a notion requires rigorous experimentation to verify. *MHRT* was initially identified as a cardiac‐specific and cardiac enriched protective lncRNA by Han *et al*. 21. It acts to protect the heart against pathological hypertrophy; yet, pathological stress such as hypertrophy and HF inhibits *MHRT* transcription in the heart 21. Recently, *MHRT* was found to suppress cardiomyocyte apoptosis induced by H_2_O_2_ to simulate the acute ischaemic condition 26, and under such context, *MHRT* expression in cardiomyocytes was activated by oxidative stress. In this same study, the authors found that the plasma *MHRT* level is markedly elevated in patients with acute MI. Nevertheless, the potential usefulness of *NRON* and *MHRT* as biomarkers of HF has not been previously evaluated.

Here, we revealed that *NRON* and *MHRT* were both elevated in their plasma levels in patients with HF relative to non‐HF control participants. We also found that these two lncRNAs are fairly abundant RNA species in human serum samples based on the relatively low Ct values of qPCR experiments (the median Ct value for *NRON* was 26.3, ranging from 22.3 to 31.4; and the median Ct value for *MHRT* was 27.0 with a range from 23.4 to 30.4 in patients with HF). These facts prompted us to propose that either of these two lncRNAs is a reasonable predictor of HF. For years, NT‐proBNP has been believed to be an established risk marker for HF. Our analysis indicated that the predictive power of *NRON* is comparable to that of NT‐proBNP: The reported value of AUC is 0.844 for NT‐proBNP 34, and the value for *NRON* was 0.865. By comparison, the AUC value for *MHRT* is lower (0.702); yet, it still falls into the ‘good’ category for clinical applications according to the guide for classifying the accuracy of a diagnostic test with the traditional academic point system (0.9–1.0 excellent; 0.8–0.9 very good; 0.7–0.8 good; 0.6–0.7 sufficient; 0.5–0.6 bad; < 0.5 test not useful) 35, 36.

In an earlier study, Kumarswamy *et al* 16. conducted global transcriptomic analyses in plasma RNA from patients with or without left ventricular remodelling after MI with three independent patient cohorts developing cardiac remodelling and HF. The authors found that *LIPCAR* is down‐regulated early after MI but up‐regulated during later stages. Plasma levels of *LIPCAR* can predict patients developing cardiac remodelling and future cardiovascular deaths. Li *et al*. 17 analysed the expression levels of lncRNAs in whole blood, tissue and plasma in a mouse model of acute HF. The study revealed that 518 lncRNAs are up‐regulated while 908 are down‐regulated in the heart with microarray‐based analyses with 32 differentially expressed lncRNAs with changes greater than twofold. Greco *et al*. 29 profiled and validated lncRNAs in left ventricle biopsies of 18 patients affected by non‐end‐stage dilated ischaemic cardiomyopathy and 17 matched controls. Fourteen lncRNAs were significantly modulated in non‐end‐stage HF patients, identifying a HF lncRNA signature. In particular, *CDKN2B‐AS1/ANRIL* (antisense non‐coding RNA in the INK4 locus), *HOTAIR* (HOX transcript antisense RNA) and *LOC285194/TUSC7* (tumour suppressor candidate 7) showed similar modulation in peripheral blood mononuclear cells and heart tissue, suggesting a potential role as disease biomarkers. Yan *et al*. 37 identified an lncRNA *UCA1* (urothelial carcinoma‐associated 1) as a biomarker for acute myocardial infarction (AMI) with its plasma level significantly decreased in AMI patients, compared with non‐AMI subjects.

In one of our previous studies, we reported two lncRNAs zinc finger antisense 1 (*ZFAS1*) and Cdr1 antisense (*CDR1AS*) as novel biomarkers of acute MI, with their reciprocal changes in the whole blood samples (*ZFAS1* was down‐regulated, whereas *CDR1AS* was up‐regulated) independently predicting acute MI 38. Intriguingly, in the context of HF as in the present study, these two lncRNAs did not show significant alterations in their circulating levels, indicating that they may be specific for predicting acute MI. To the best of our knowledge, there have been no other published studies on the circulating lncRNAs in HF patients. Our study therefore represents the first of such efforts to identify biomarkers with the potential to predict HF in humans.

### Significance of our findings

*NRON* and *MHRT* as biomarkers could offer a number of advantages. First, lncRNAs have been found to be overall more stable than protein markers in circulation and can be easily detected in blood samples (whole blood, plasma and serum); thus, it is possible that *NRON* and *MHRT* might also be more stable in the blood than the traditional protein markers 15, 39, 40. Second, *NRON* and *MHRT* can be detected in a quantitative manner by highly sensitive methods such as real‐time PCR. And finally, changes in *NRON* and *MHRT* in the bloodstream may reflect alterations of cardiac function and structure during the development of heart disease thereby helping us to infer the underlying molecular mechanisms. This is in resembling miRNAs as biomarkers of heart disease. For example, the early elevation of circulating *miR‐1* during acute myocardial infarction can be interpreted as increased apoptotic cardiomyocyte death 41, 42, 43.

### Limitations of our study

In the present study, we focused on only a subset of lncRNAs that are known (at the time we initiated our study) to be relevant to cardiac disease without dealing with the global transcriptome profiling. Thus, our findings do not provide a panorama for comprehensive understanding of all lncRNAs identified thus far, but might have missed out many other important lncRNAs that were not included in the present study for their potential as HF biomarkers. Nonetheless, the lncRNAs selected for our study are those that have been shown to be able to cause cardiac disorders or are abundantly expressed in heart cells. Another limitation of the study is the unknown sources of *NRON* and *MHRT* in the bloodstream: Are they released from dead cells in the failing heart or are they secreted by blood cells in response to the damaged heart?

## Conflict of interest

None declared.

## Author contributions

LNX, LHS, YZ, YJL and BFY designed, performed study and supervised all aspects of the research and analysis. LNX, YJL and BFY finalized the manuscript. LNX, LHS, YZ, YCH, YH, QQL, YG, BBF, LNC, XXW, CQX, MYZ and ZGW assisted in research, data analysis and interpretation. BY, YT, SW, ZMD and YJL were responsible for collect blood samples and for the final approval of the manuscript. YH was responsible for the statistical analysis involved in the study. BY, YT, SW and ZMD reviewed the clinical aspects and writing of manuscript.

## Supporting information


**Table S1** The demographic characteristics and HF‐relevant indicators in HF patients, non‐HF control participants for *NRON*
**Table S2** The demographic characteristics and HF‐relevant indicators in HF patients, non‐HF control participants for *MHRT*
**Table S3** The Statistical Analysis of Circulating *NRON* and *MHRT*
**Table S4** Human gene‐specific primers for real‐time PCR
