# Supplementary material for: Circulating long non‐coding RNAs NRON and MHRT as novel predictive biomarkers of heart failure
Source: J Cell Mol Med. 2017 Mar 14;21(9):1803–14. doi: 10.1111/jcmm.13101 (PMC5571539; doi:10.1111/jcmm.13101)
Supplement: Supplementary file 1 — Table S1 The demographic characteristics and HF‐relevant indicators in HF patients, non‐HF control participants for NRON Table S2 The demographic characteristics and HF‐relevant indicators in HF patients, non‐HF control participants for MHRT Table S3 The Statistical Analysis of Circulating NRON and MHRT Table S4 Human gene‐specific primers for real‐time PCR [file JCMM-21-1803-s001.doc]

**Circulating Long Non-Coding RNAs *NRON* and *MHRT* as Novel Predictive Biomarkers of Heart Failure**

Lina Xuan^1^, Lihua Sun^1^, Ying Zhang^1^, Yuechao Huang^1^,Yan Hou^2^, Qingqi Li^1^, Ying Guo^1^, Bingbing Feng^1^, Lina Cui^1^, Xiaoxue Wang^1^, Zhiguo Wang^1^, Ye Tian^3,4^, Bo Yu^5^, Shu Wang^3^, Chaoqian Xu^1^, Mingyu Zhang^1^, Zhimin Du^6^, Yanjie Lu^1^,*, Baofeng Yang^1,7^*

^1^Department of Pharmacology, Harbin Medical University (the State-Province Key Laboratories of Biomedicine-Pharmaceutics of China, Key Laboratory of Cardiovascular Research, Ministry of Education), College of Pharmacy, Harbin Medical University.

^2^Department of Epidemiology and Biostatistics, Public Health School, Harbin Medical University.

^3^Department of Cardiology, the First Affiliated Hospital, Harbin Medical University.

^4^Division of Pathophysiology (the State-Province Key Laboratories of Biomedicine-Pharmaceutics of China and the Key Laboratory of Cardiovascular Research, Ministry of Education), Harbin Medical University.

^5^Department of Cardiology, the Second Affiliated Hospital, Harbin Medical University;

^6^Institute of Clinical Pharmacology, the Second Affiliated Hospital, Harbin Medical University, Harbin, Heilongjiang, China.

^7^Department of Pharmacology and Therapeutics, Melbourne School of Biomedical Sciences, Faculty of Medicine, Dentistry and Health Sciences, University of Melbourne, Melbourne, Australia.

*Corresponding

Bao-Feng Yang: e-mail: [yangbf@ems.hrbmu.edu.cn](mailto:yangbf@ems.hrbmu.edu.cn) or Yanjie Lu: yjlu2008@163.com;

Postal address: Department of Pharmacology (the State-Province Key Laboratories of Biomedicine-Pharmaceutics of China), Harbin Medical University, 157 Baojian Road, Nangang District, Harbin, Heilongjiang, China 150081; Tel.: +86 451 8667-1354; Fax: +86 451 8667-1354.

**Supplementary Methods**

**Participants**

Between February 2014 and January 2015, 104 HF patients and 109 non-HF control subjects presented to the First Affiliated Hospital, the Second Affiliated Hospital, Third Affiliated Hospital, Fourth Affiliated Hospital of Harbin Medical University (Harbin, China). Subjects were classified as HF cases when they met the Framingham criteria for the diagnosis and if circulating NT-proBNP were above 1000 ng/L. Subjects were classified as non-HF cases if clinical diagnosis excluded HF and the circulating NT-proBNP was below the age-related cutoff points published by Januzzi *et al*. The study protocols were procured in accordance with the guidelines of and approved by the Ethics Committee of the Harbin Medical University. The clinical characteristics of the study population are summarized in **Table 1**.

**Collection and Handling of Human Blood Samples**

Whole blood (WB) samples (1 mL per patient) were drawn from the study subjects via a direct venous puncture into tubes containing sodium citrate. The human whole blood samples in sodium citrate vacuum tubes was kept at 4°C and then centrifuged at 3000 rpm/min at 4^o^C for 10 min to obtain plasma samples.

**Quantitative Real-Time Reverse Transcription (RT)-Polymerase Chain Reaction (PCR)**

Total RNA was isolated from 0.5mL plasma sample using phenol/chloroform extraction procedures as described before. The first-strand cDNA was synthesized using the Reverse Transcription System (Promega) according to the manufacturer’s instructions as described previously. The SYBR Green PCR Master Mix Kit (Applied Biosystems) was used in real-time PCR for relative quantification of lncRNAs. PCR was performed on 7500 FAST Real-Time PCR System (Applied Biosystems). The PCR primer pairs are listed in **Table S4**. Relative expression of lncRNAs was calculated using the comparative cycle threshold (Ct) method (2^-ΔΔCt^). LncRNA levels were normalized to GAPDH as the internal control for both inter-well and inter-RNA sample variations. The final results are expressed as fold changes by normalizing the test values to the non-HF control values.

**Statistical Analysis**

Categorical data were presented with count and percentile. Continuous variables were described as mean ± SEM (standard error of measurement), min, max, median and interquartile range. Student t test was used to compare the demographic and clinical pathological characteristics between HF patients and non-HF subjects when data follows normal distribution, otherwise Wilcoxon rank sum test was used. Chi-squares were used to analyze the categorical data between two groups. Wilcoxon rank sum was performed to compare the expression of lncRNAs between HF patients and non-HF subjects. Univariable and multivariable logistic regression analyses were conducted to evaluate whether the lncRNAs are the independent factors for HF. Univariate is used assuming that the response variable is influenced by only one other factor. Multivariate analysis is used to describe analyses of data where there are multiple variables or observations that may be are interrelated for each unit or individual. Thus, multivariate analysis considers the relationship between response variable and one other factor controlling for other variables. The area under Receiver operator characteristic (ROC) curve (AUC) was used to evaluate the predictive power of circulating lncRNA levels for AMI. Spearman rank correlations were used to evaluate the association between levels of lncRNAs and cardiac risk factors, conventional AMI markers, and cardiac function parameters. All analyses were carried out with SAS 9.1 (Serial No. 989155) except that ROC was done with SPSS v17.0 software. The significant level was set at 0.05 and two-tailed P values <0.05 were considered statistically significant.

**References**

1. **Jae-Hyung L, Chen G, Guangdun P, Christopher G, Shuxun R, Yibin W, Xinshu X.** Analysis of transcriptome complexity through RNA sequencing in normal and failing murine hearts. *Circulation Research*. 2011; 109: 1332-41.

2. **Tijsen AJ, Creemers EEMoerland PD, de Windt LJ, Ac VDW, Kok WE, Pinto YM.** MiR423-5p as a circulating biomarker for heart failure. *Circulation Research*. 2010; 106: 1035-9.

3. **Jing A, Rong Z, Yue L, Jielin P, Yanjie L, Jundong J, Kang L, Bo Y, Zhuqin L, Rongrong W.** Circulating microRNA-1 as a potential novel biomarker for acute myocardial infarction. *Biochemical & Biophysical Research Communications*. 2010; 391: 73-7.
